# Ecological drift simulations reveal key factors influencing minimal microbiome engineering and community assembly

**DOI:** 10.1093/ismeco/ycag067

**Published:** 2026-03-21

**Authors:** Silvia Talavera-Marcos, Daniel Aguirre de Cárcer

**Affiliations:** Microbial and Environmental Genomics Group, Departamento de Biología, Universidad Autónoma de Madrid, Madrid 28049, Spain; Microbial and Environmental Genomics Group, Departamento de Biología, Universidad Autónoma de Madrid, Madrid 28049, Spain

**Keywords:** minimal microbiomes, ecological drift, simulation modeling

## Abstract

In this work, we describe an engineering approach that leverages ecological drift to generate minimal microbiomes; microbial consortia that are relatively simple, cohesive, and functionally complete. This process can be applied to any microbial ecosystem, provided that the target microbiome can be experimentally mimicked. Empirical support for this approach has emerged from multiple independent studies. We use simulations across diverse scenarios, significantly varying niche structures and biotic interactions, to explore the experimental conditions and source microbiome characteristics that favor successful outcomes, within a computational framework that also enables the study of microbial community assembly. Our results indicate that the effectiveness of this approach is constrained by several factors, and that perfect outcomes should not be routinely expected. Nevertheless, despite its drawbacks, this strategy remains a powerful tool for simplifying microbiomes and isolating key co-adapted populations, enabling the construction of low-diversity consortia that retain community function and present ecological cohesion.

## Graphical Abstract

**Graphical Abstract:**
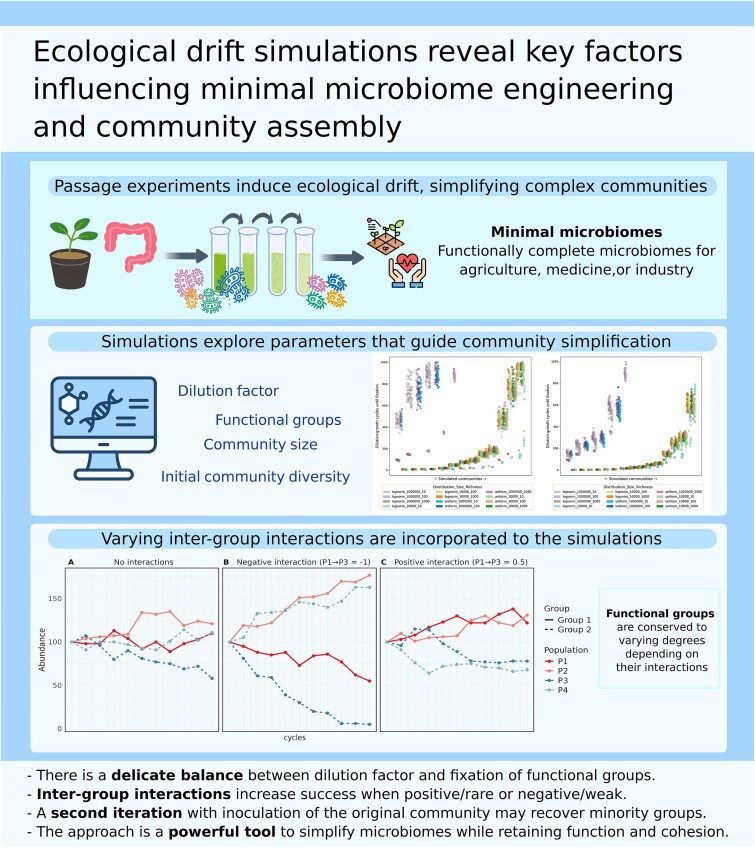

## Introduction

The domestication of microbiomes is a major goal in modern biology [1], with significant implications for agriculture, medicine, and biotechnology [2]. Small, engineered synthetic consortia have been able to carry out functions such as bioremediation [3], manipulating plant phenotypes [4], or biofuel production [5], among others. Notwithstanding these stories of success, the bottom–up design of functional microbiomes remains challenging. This approach, selecting the right microbial populations to build a functional synthetic community, often requires a deeper understanding of specific microbial ecosystems, as well as microbial ecology and function in general, than we currently possess. In an attempt to partially circumvent this fundamental knowledge gap, several groups have pursued the rational design of synthetic communities using computational approaches based on mechanistic ecological modeling [6–8] and metabolic network modeling [9–11]. Nevertheless, recognizing that this limitation cannot be fully resolved by predictive modeling alone, Chang *et al.* introduced the idea that, rather than fighting unavoidable biological forces, an alternative engineering approach could harness eco-evolutionary forces to create effective microbial consortia [1].

Attempts to remediate dysbiotic states in host-associated microbiomes (studies thus far overwhelmingly circumscribed to mammals and plants) using single bacterial populations have not been too successful. The scientific consensus nowadays is that complex consortia should be used instead of single strains, so that they can comprehensively replace the resident dysbiotic microbiota. The same is true for plant inoculants; single populations can seldom withstand the encounter with the rich reservoir represented by the native soil microorganisms. The key question then relates to how these complex communities should be constructed.

Many approaches have relied on rather observational information, constructing synthetic communities on the grounds of the taxonomic composition of “healthy” microbiomes using a library of isolates (a bottom–up approach). However, these approaches have so far provided limited success. For instance, in their seminal work, Ridaura *et al.* [12] were able to modify the obese phenotype in mice using both co-housing with a lean mate and fecal transplantation experiments, but could not repeat the results using a specifically tailored consortium of 39 strains representative of the lean microbiota.

Microbial ecosystems most often consist of patches of strongly interacting dense microbial consortia [13], termed “local communities”. Within these microscale patches, the short distances between cells enable efficient diffusion-based exchange of metabolites, fostering strong biotic interactions that significantly shape the structure and dynamics of the community. Thus, populations that compete or antagonize one another may exclude each other within a single patch, but can still co-exist across the broader ecosystem when all patches (i.e. local communities) are considered collectively. Similarly, only co-adapted population pairs are able to inhabit the same patch, yet multiple such pairs can co-exist within a larger (macroscale) sample taken from the ecosystem.

Inevitably, the scale of most microbiological samples far exceeds that of the local community. This “Higher-scale sampling bias” [14], in which samples represent composites of multiple local communities, makes it extremely challenging to isolate co-adapted populations. In line with this rationale, the failure of Ridaura *et al.*’s bottom–up approach may have resulted from a lack of co-adaptation among the selected strains. Therefore, how can we effectively bypass the higher-scale sampling bias to isolate cohesive and functionally complete microbial communities? An elegant yet untried solution is to actively manipulate ecological drift, defined as the stochastic changes in the relative abundance of populations in a community over time.

The cornerstone of the approach explored here, is a process (“the process” from now on) in which ecological drift is experimentally manipulated to isolate and reconstruct cohesive, local-like communities from complex microbial ecosystems. The underlying idea is that local communities represent functionally complete and cohesive microbial consortia. The overall idea of the process is relatively simple; microbial ecosystems consist of numerous microscale patches, within which individual local communities assemble. In a passage experiment, a sample (representing a macroscale portion of the ecosystem) is inoculated into a new, sterile environment. Bacteria are allowed to colonize this new environment, and the process is repeated multiple times. If the number of passages, colonization duration, and dilution rates between passages are properly calibrated, ecological drift should cause all local communities to eventually converge to a single community composition. Importantly, the final selected communities are not necessarily expected to correspond to intact local communities originally present in the source ecosystem. Instead, they may emerge from the stochastic loss, retention and reassembly of populations originating from multiple local communities, giving rise to novel but cohesive consortia during the serial passage process.

In most microbial ecosystems, the numerous microscale patches available to bacteria will fall into a limited number of categories, based on their abiotic conditions and (or) host-derived biotic environments in the case of host-associated microbiomes (e.g. normal epithelial patches vs. cecal crypts in the intestine). As a result, the process should yield homogeneous local community compositions for each type of patch. In this context, bacteria sampled from the resulting experimental ecosystem are likely to be co-adapted and capable of occupying all available niches within the ecosystem. Consequently, the resulting consortium could be considered functionally complete and cohesive. Naturally, the resulting microbiome will exhibit a significantly reduced richness (i.e. number of distinct microbial populations) compared to a typical sample from the same natural ecosystem, which would contain many different local community compositions across patch types. Therefore, generating a representative isolates library from the drift-simplified microbiome would entail substantially lower isolation costs compared to doing so from a natural, more complex sample.

Empirical support for the process has emerged in multiple independent studies. For instance, Goldford *et al.* [15] investigated microbial community assembly using a single carbon and energy source. By cultivating complex microbial communities *ex situ*, derived from diverse natural environments, they observed that, for each compound, communities assembled into highly variable compositions at the finest phylogenetic resolution analyzed [i.e. exact sequence variants (ESVs) of the 16S rRNA gene]. Despite this variability at the ESV level, communities consistently converged on the same family-level composition for each carbon source, regardless of their distinct environmental origins. In all passage experiments, the final communities were dominated by two populations, one from each of two bacterial families. However, different final communities harbored different inter-family pairs, indicating that all endpoint communities were composed of distinct homogenous, local-like cohesive consortia. Notably, these experiments were performed in spatially well-mixed systems, and therefore reflect the emergence of functionally cohesive local-like communities rather than spatially structured local communities sensu metacommunity theory.

Second, Morella *et al.* conducted a natural passage experiment on tomato phyllosphere microbiomes [16], a spatially structured ecosystem composed of multiple heterogeneous local communities. Despite this spatial complexity, successive passaging resulted in a marked decline in richness and in the emergence of communities that were resistant to invasion by the original microbiota. These outcomes are fully consistent with the predictions of the drift-based process described here, and support the notion that drift-driven simplification can generate cohesive and functionally complete consortia not only in well-mixed systems, but also in spatially structured metacommunities.

The process, therefore, may serve as a valuable method for generating Minimal Microbiomes, microbial consortia that are relatively simple, cohesive, and functionally complete, from any microbial ecosystem, provided that the ecosystem can be experimentally mimicked and subjected to a passage experiment. The ability to obtain Minimal Microbiomes holds significant potential to drive the anticipated technological and economic advances in the field. These consortia could serve either as final products (with or without selection for specific community-level phenotypes) or as foundational materials for engineering genetically modified consortia, depending on the application area and, crucially, the intended end use. Although the resulting minimal consortia are expected to remain functionally complete, they are not necessarily expected to exhibit enhanced functional performance. Rather, they should be viewed as simplified and cohesive starting points from which subsequent engineering or selection could further improve functional performance.

From an ecological perspective, several lines of evidence indicate that microbial communities often exhibit a coarse-grained functional structure in which subsets of populations perform equivalent ecological roles, a pattern that has been repeatedly observed across diverse experimental and natural microbiomes [15, 17, 18]. Biotic interactions among populations belonging to the same microbial community, including facilitation and antagonism, have also been repeatedly observed across experimental and natural systems [7, 19, 20]. Within each functional group, stochastic demographic processes can influence the dominance of individual populations, whereas between functional groups, biotic interactions could shape community stability and composition. Importantly, recent work has shown that microbial community assembly often converges to reproducible functional structures despite taxonomic variability, reflecting strong functional constraints and inter-group interactions [18, 21]. In this context, drift-driven simplification can be viewed as a process acting on a functionally structured community, where fixation, extinction and inter-group interactions jointly determine the emergence of minimal yet cohesive consortia.

The overall goal of this study is to assess the feasibility of the proposed process for generating Minimal Microbiomes, and to explore under which experimental conditions and source microbiome characteristics might be successful. To this end, we developed a computational framework that reproduces dilution–growth dynamics and simulates the final composition of microbial communities based on their initial characteristics and experimental parameters. Importantly, the simulation framework implemented here explicitly models well-mixed communities, and therefore captures the assembly and stabilization dynamics of local-like microbial consortia rather than spatially structured metacommunities sensu metacommunity theory. Accordingly, the framework does not attempt to reproduce the spatial structure of natural microbial ecosystems. Nevertheless, we posit that ecological drift constitutes a fundamental driver of community assembly both within individual local communities and across spatially structured metacommunities. The conceptual formulation of the proposed approach explicitly assumes that natural microbial ecosystems are composed of multiple spatially structured local communities, and that the drift-driven process described above operates within this ecological context. While explicitly modeling spatial heterogeneity would require substantially more complex formulations, the present framework focuses on the minimal, well-mixed unit of community assembly. Although this simplified representation does not reproduce the full spatial organization of natural systems, it provides a tractable framework to explore the general dynamics underlying the proposed process. From this perspective, the results presented here provide general insights into how drift can be exploited to generate minimal, cohesive and functionally complete consortia in experimental systems, while still being informative for understanding similar processes in heterogeneous natural metacommunities.

Beyond its application to microbiome engineering, this framework also provides a controlled setting to study the ecological assembly of microbial communities, allowing us to explore how community structure emerges from population-level traits, stochasticity, functional redundancy and inter-population interactions. We then used this toolkit to identify how those characteristics relate to the parameter settings that would be required to achieve fixation of non-redundant populations in a real-world passage experiment. To address this goal, we employed three simulation scenarios of increasing complexity, each progressively incorporating the following features: (i) The presence of functionally redundant populations and stochastic growth. (ii) The existence of functional groups with fixed relative abundances in the ecosystem and intra-group diversity. (iii) The presence of interactions between populations belonging to different functional groups. Importantly, by explicitly modeling dilution-driven stochastic loss, functional redundancy and inter-population interactions, this framework can be interpreted as a general experimental model for studying microbial community assembly under strong ecological drift.

## Materials and methods

### Reanalysis of published serial passaging microbiome data

To evaluate whether patterns predicted by our framework are consistent with empirical observations, we reanalyzed publicly available 16S rRNA sequencing data from the previously mentioned serial passaging experiments of Goldford *et al.* Sequence filtering, trimming, ASV inference, merging of paired reads, and chimera removal were performed using the DADA2 R package following the workflow described by the original authors. Given that the sequence identity of most intragenomic 16S rRNA gene sequences typically differs by less than 1%, sequences were clustered against the Greengenes v13.5 99% reference dataset and reference trees. To account for varying sequencing efforts, samples were subsampled to a uniform depth of 4380 counts per sample based on OTU abundances prior to richness estimation and figure generation.

### Simulating initial communities and dilution-growth cycles

The first step is to generate simulated initial communities with varying characteristics. Abundance tables are used where each column represents a community and each row represents a population. The characteristics explored throughout this work include: the number of distinct populations, the total community size (i.e. number of individuals) and the distribution of population abundances. The abundance of each population is sampled randomly from a uniform or log-normal abundance distribution.

**Figure 1:**
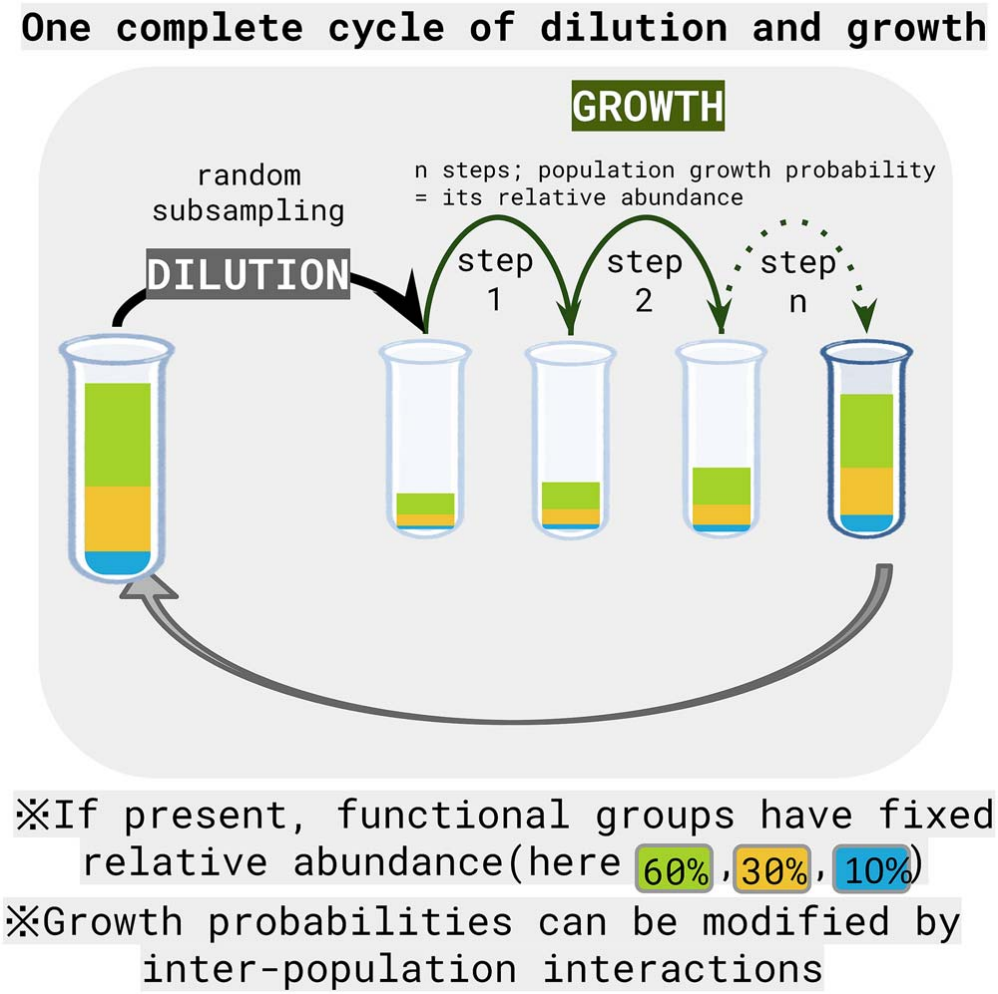
Diagram depicting the simulated dilution and growth process**.**

The next step is to simulate the dilution–growth process (Fig. 1, Table 1), where several parameters must be specified, such as the number of replicated trajectories per initial community, the number of dilution–growth cycles, and the dilution factor employed. Each trajectory thus represents a single initial simulated community (i.e. the sum of all populations) undergoing a number of dilution–growth cycles. During each cycle, the dilution step is simulated by sampling the community with replacement to the desired final community size and then allowed to grow back to its original size. The dilution step thus reduces the original community size by the dilution factor (D).

**Table 1:** Glossary.

Carrying capacity	The maximum size of a population or group of populations that a given environment is capable of sustaining based on the resources it contains. In the context of this work, carrying capacities define the niche sizes for functional groups, determining the maximum abundance each group can reach during growth phases.
Community	A set of populations that coexist in space and time and have the capacity to interact with each other, forming a living system with its own structure, relationships with the environment, and functions^,^. In the context of this work, communities are simulated as sets of unique populations that may or may not belong to different functional groups and can potentially have interactions between each other.
Dilution-growth cycle	A complete cycle consisting of a dilution step that reduces community size by the dilution factor, and a growth phase where the diluted community grows back to the target total abundance through multiple growth iterations. In this work, multiple cycles are simulated to observe fixation dynamics.
Ecological drift	Random changes in the relative abundances of populations in a community. This is the key force modeled in the simulations, where stochastic sampling during dilution and growth phases leads to changes in community composition over time.
Fixation	In this work, fixation is said to occur when a single population becomes dominant within its functional group through the dilution-growth process.
Fixation threshold	The relative abundance value that a population must exceed to be considered fixed within its functional group. In this work, fixation thresholds of 50% and 90% are used to define successful fixations.
Fixed population	A population that has reached fixation, meaning it has exceeded the fixation threshold and is the only population remaining (or dominant) in its functional group.
Functional group	In natural communities, the presence of at least one population of each functional group would be necessary for maintaining the community’s functional stability. In this work, a functional group represents a set of populations that share the same niche within a community; each group grows separately until reaching its respective carrying capacity, with stochastic neutral growth within groups.
Microbial population	A set of microorganisms that are homogeneous genetically and functionally and coexist in the same local community. Microorganisms in a population share the same properties and distinctive dynamics, which produce high ecological cohesion of the group.
Success rate	The percentage of the 100 simulated trajectories of the same initial community in which a population reaches fixation for each functional group.
Success threshold	The minimum success rate for a dilution-growth experiment to be considered successful.

Growth is simulated through multiple growth iterations within each cycle. In each iteration (*k*), the growth probability for a population *i* is equivalent to its current relative abundance. If *N_i_* is the number of individuals of *i*, the probability that additional individuals are assigned to population *i* in iteration *k*:


$${p}_i(k)=\frac{N_i\left(k-1\right)}{N_{total}\left(k-1\right)}$$

The growth magnitude (*I_k_*) varies from iteration to iteration based on a fixed portion of the community size. For example, for a growth magnitude set at 1%:


$${I}_k=0.01\times{N}_{total}\left(k-1\right)$$

We chose this strategy instead of simply adding a single individual at each growth iteration to speed up the computation.

If functional groups are present in the simulation (see scenarios 2 and 3 below), factors such as the number of functional groups, the assignment of populations to these groups and the relative abundance of each group (i.e. its niche size) need to be taken into account. In this case, the abovementioned stochastic growth occurs within each functional group, and all groups grow separately until reaching their carrying capacities (*K*) (i.e. niche sizes). Let *G* be the functional group to which population *i* belongs. The conditional selection probability for population *i*, given the *K_G_* assigned to its group, is proportional to its relative abundance within that group:


$${P}_{i\mid G}(k)=\frac{N_i\left(k-1\right)}{N_G\left(k-1\right)}$$

In order to keep the niche sizes stable over iterations, the magnitude of growth of each group, *I_k|G_*, is weighted by its carrying capacity:


$${I}_{k\mid G}={I}_k\times{K}_G$$

The presence of biotic interactions (see scenario 3 below) modifies these probabilities by considering an interaction matrix (*A*). This table is multiplied by the probability vector *p(k)* (which includes the *p_i_* values), and the result is added back to the original probability vector. Thus, the modified vector *p′* for a single iteration is:


$${p}^{\prime }(k)=p(k)+A\cdotp p(k)$$

Hence, for a single population *i*, the growth probability is calculated as the sum of multiple components: its own relative abundance and the relative abundances of the populations it interacts with, weighted by the strength (positive or negative) of each interaction:


$$p{\prime}_i(k)={p}_i(k)+\sum{A}_{ij}{p}_j(k)$$

Where *A_ij_* is the effect of population *j* on population *i* (i.e. the corresponding value in the interaction matrix; see Supplementary Fig. 2).

The final community composition at the end of each dilution–growth cycle is stored for downstream analysis.

To process and interpret the results, we define two key parameters: the success threshold and the fixation threshold. The fixation threshold is the minimum relative abundance (typically 50% or higher) required for a population to be considered fixed within its functional group in a given trajectory. On the other hand, a simulation experiment is considered successful when a fixed population appears in each functional group in at least a given percentage (i.e. the success threshold) of its replicated trajectories. For example, let us consider a simulated dilution–growth experiment with a fixation threshold of 50% and a success threshold of 95%. For each unique initial community being analyzed, 100 independent simulations (trajectories) are performed. In some trajectories, a single population may reach a relative abundance greater than 50% as early as cycle 20, while in others this may not occur until cycle 25. However, if by cycle 30, 95% of the trajectories show fixation of a single population (i.e. a relative abundance >50%) for each functional group, then we would consider that, under these fixation and success thresholds, the experiment reaches success at cycle 30. It is important to note that success does not require the same population to be fixed across trajectories.

**Figure 2:**
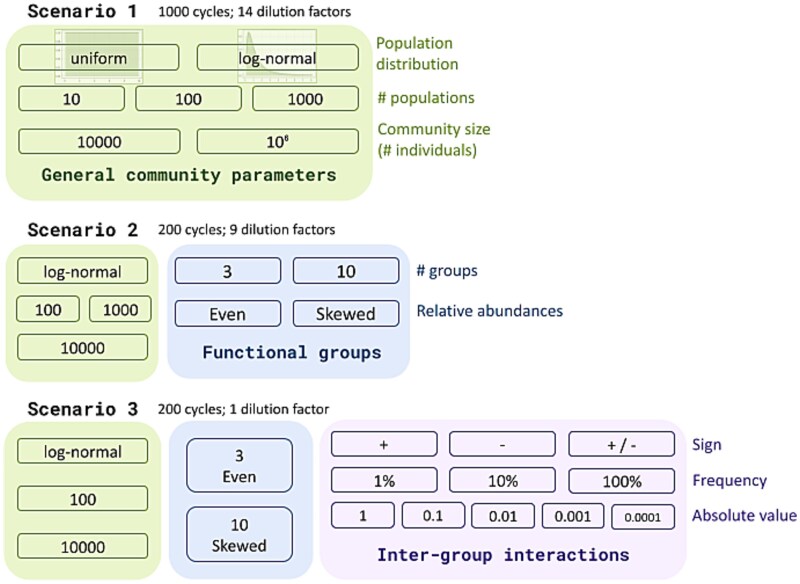
Diagram summarizing the characteristics of the three simulation scenarios explored.

**Scenario 1.** For this opening exploration (Fig. 2), we generated a set of initial communities by combining three parameters: richness (10, 100, or 1000 distinct populations), community size (10^4^ or 10^6^ individuals), and abundance distribution (uniform or log-normal). This resulted in 12 unique parameter combinations. For each unique combination, we constructed 30 replicate communities, yielding a total of 360 different initial communities. Each of the 360 initial communities was subjected to a series of simulated dilution-growth experiments, using one of 14 different dilution factors: 0.00025, 0.0004, 0.0005, 0.001, 0.0025, 0.004, 0.005, 0.008, 0.01, 0.025, 0.04, 0.05, 0.1, and 0.25. This resulted in a total of 5040 distinct simulated experiments. Moreover, each experiment was independently replicated 100 times, yielding 504 000 individual simulation trajectories. Each trajectory consisted of 200 dilution-growth cycles, using a growth rate of 1%. In the analysis of the results, a 95% success threshold was applied, along with two alternative fixation thresholds set at 50% and 90%.

To quantify the effects observed during the graphical exploration of results, we modeled success by incorporating all relevant parameters using a random forest regressor implemented with the *party* package in R using 1000 trees. To this end, we applied the Box-Cox transformation to the target variable (success) to approximate a normal distribution and thus help balance the data and ensure that the model performs with comparable accuracy across the full range of target values.

**Scenario 2.** Here we established functional groups with a fixed carrying capacity and stochastic growth of populations within each group. As in Scenario 1, we used 30 randomly generated communities for each community type. However, given the increased complexity of the simulations and based on the results from the previous scenario, we reduced the total number of parameter values explored. We therefore restricted our analysis to communities consisting of 10 000 individuals, with log-normal abundance distributions and initial richness levels of either 100 or 1000. As a result, the number of distinct community types was reduced from twelve to two, defined solely by initial richness. Accordingly, the total number of initial communities decreased from 360 to 60. We analyzed nine different dilution factors (0.00025, 0.0005, 0.001, 0.0025, 0.005, 0.01, 0.025, 0.05, and 0.1) instead of 14, over 200 dilution-growth cycles. The success threshold remained at 95%, and the fixation threshold studied was 50%.

To incorporate the existence of functional groups, we used three or ten functional groups with either homogeneous or heterogeneous niche sizes, resulting in four different configurations (3 or 10 groups; homogeneous or heterogeneous niche sizes). The growth rate of each group was proportional to its niche size, with 1% growth per step distributed among the populations of each group according to their group’s niche size.

**Scenario 3.** In this case, we explored the potential effect of inter-group interactions (i.e. between populations from different functional groups) on the overall success of the strategy, as well as on the fixation and extinction of functional groups. To do this, we reduced the number of communities studied to a single community from the previous scenario; one of the 30 communities with a log-normal abundance distribution and richness of 100. We analyzed the behavior of (i) 3 groups with equal niche sizes and (ii) 10 groups with heterogeneous niche sizes. For each community, 100 simulation runs were performed with 200 dilution-growth cycles at a single dilution factor: 0.1, the mildest dilution tested. This value was chosen because, in the previous scenario, success rates ranged from 0% to 95%, allowing for both potential increases and decreases in success rates, thereby revealing possible positive or negative effects of inter-group interactions.

We tested multiple types of interaction matrices, each defined by a unique combination of the following three parameters: (i) sign (positive, negative, or both), (ii) absolute value (1, 0.1, 0.01, 0.001, 0.0001), and (iii) frequency (1%, 10%, 100%). The interaction frequency refers to the percentage of possible inter-group pairs, among all distinct pairs, that exhibit an interaction. All interactions were unidirectional. Each of the 100 simulations used a different randomly generated interaction matrix sharing one specific combination of these parameter values.

The simulations are performed via C++ functions contained within the *dilgrowth* R package (available at github.com/silvtal/dilgrowth). More details about the mechanisms behind these functions are available at the Supplementary Methods. The specific scripts used for implementing the simulations and analyses are available on the GitHub repository github.com/silvtal/predicting_fixation. GPT-4 was used via the Cursor API to assist with cosmetic adjustments of plots (e.g. colors and layout).

## Results

Our conceptual framework predicts that repeated dilution–growth cycles dominated by ecological drift should progressively reduce community richness as populations are stochastically lost across transfers. Consistent with this expectation, serial passaging experiments in plant phyllosphere microbiomes have previously reported a marked decline in richness during transfers [16]. To examine whether a similar pattern is also present in other passaging experiments, we reanalyzed publicly available 16S rRNA sequencing data from Goldford *et al.* [15]. Although richness dynamics were not explicitly analyzed in the original study, our reanalysis revealed a progressive decline in OTU richness during early transfer cycles followed by stabilization at later stages (Supplementary Fig. 1). This pattern is consistent with the richness dynamics expected under repeated dilution–growth cycles dominated by ecological drift.

**Figure 3:**
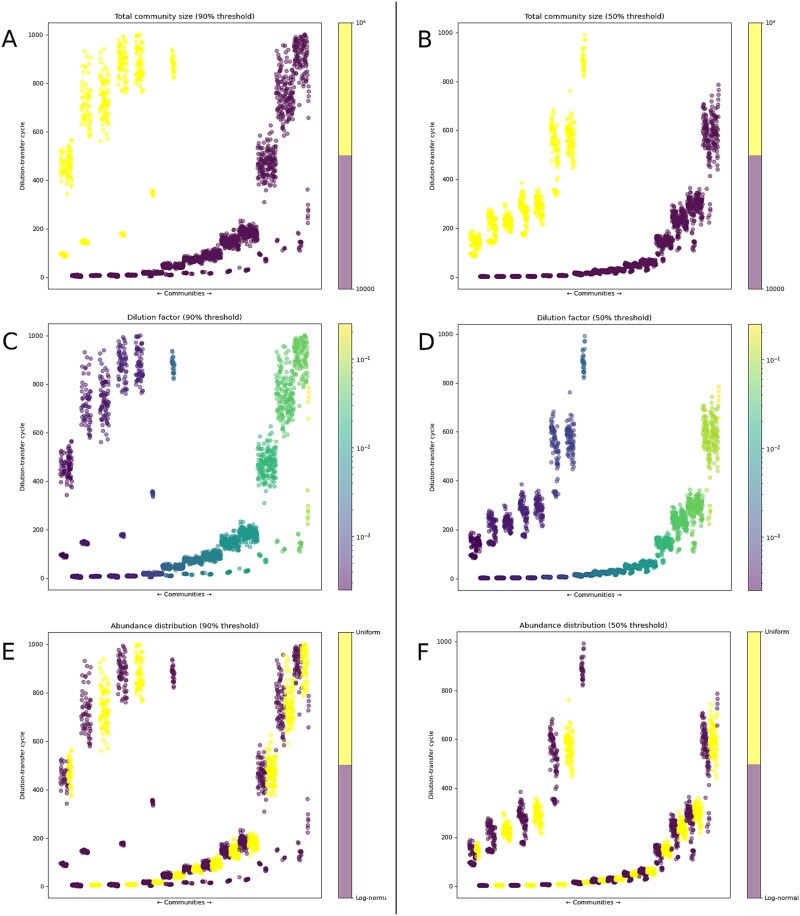
Success patterns of dilution-growth processes according to the simulation variables used. The *Y* axis indicates the dilution-growth cycle in which success occurred, while the *X* axis orders the different simulated communities by dilution factor, distribution, community size, and richness. In each plot, the simulations are colored according to one of the following variables, from top to bottom: community size (A and B), dilution factor (C and D), and population distribution (E and F). The plots show success according to two fixation thresholds: 90% in the first column and 50% in the second one. The success threshold is 95% in both cases.

Our initial simulation scenario was designed to explore the effect of the different experimental parameters (community richness, size and distribution, and dilution factor) on success, defined in this initial scenario as the fixation of a single population within the community (Fig. 3, Supplementary Fig. 3). First, we observed a strong effect of community size, with smaller communities reaching success more rapidly (Fig. 3A and B). Second, there is a clear trend related to the dilution factor: within each community size, success is achieved earlier as dilution strength increases (Fig. 3C and D). In contrast, the abundance distribution appears to have little impact in most cases (Fig. 3E and F) as did richness or diversity metrics (Supplementary Fig. 3). We also noted that dilution factors above 0.1 or 0.25 (for 90% and 50% fixation thresholds, respectively) did not lead to successful outcomes, particularly for larger communities. Therefore, these dilution factors were excluded from subsequent analyses in this scenario, as well as from simulations in subsequent scenarios.

To quantify the effects observed during the graphical exploration, we constructed a random forest model. The model evaluates the importance of each variable by measuring the decrease in model accuracy when the values of that variable are randomly permuted. Variables whose permutation leads to a greater reduction in accuracy are considered more important. Consistent with the graphical observations, the model identified community size, dilution factor, and diversity metrics as the most influential variables, in that order (Table 2). However, recognizing that the inclusion of multiple diversity metrics introduces redundancy, we compared several new models, each including only one diversity measure, with or without richness as an additional variable. The results, based on the mean decrease in accuracy for each variable (Supplementary Tables 1 and 2) and the R^2^ values associated with each model (Supplementary Table 3), confirmed that the diversity metrics generally had a much weaker effect compared to community size and dilution factor. Among the diversity metrics, the Shannon diversity index exhibited marginally higher predictive power.

**Table 2:** Feature importance in the random forest model. Feature importance is calculated based on the mean decrease in accuracy. The values indicate how much the model’s accuracy drops when the variables are randomly permuted, for different fixation thresholds (50% and 90%).

Fixation threshold	Community size	Dilution factor	Shannon diversity	Gini index	Pielou’s evenness	Abundance distribution	Richness
50%	0.545	0.485	0.043	0.036	0.028	0.022	0.014
90%	0.519	0.501	0.053	0.082	0.048	0.051	0.035

**Figure 4:**
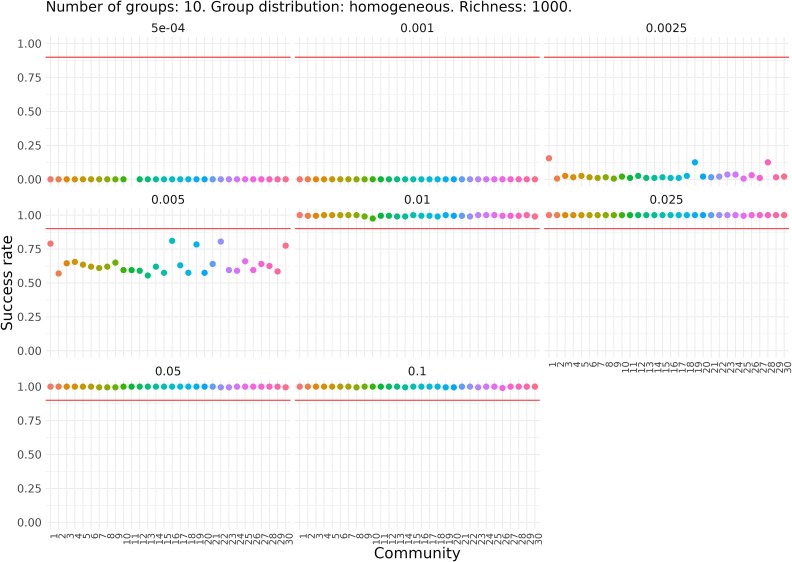
Success rate for each of the 30 simulated communities with 10 functional groups, homogeneous relative abundances, and a richness of 1000. Each plot corresponds to a different dilution factor. The *Y* axis indicates the proportion (from 0 to 1) of dilution-growth simulations in which total success occurs; that is, fixation in all functional groups. Each point represents one of the 30 communities. The missing point corresponds to a community that, after undergoing extinctions and a drop in total abundance, could no longer be sustained under the applied dilution factor.

Next, we explored the success of the approach with simulated communities formed by 3 or 10 functional groups, each with its own fixed relative abundance and stochastic growth of intra-group populations. We observed that communities with 10 functional groups and heterogeneous relative abundances never achieved full success at any dilution factor, regardless of their richness (Supplementary Figs 4 and 5). This is expected since some groups have very low relative abundances (as low as 0.1%), making them highly vulnerable to extinction at each dilution step, even with milder dilution factors. In contrast, communities with 10 groups and homogeneous relative abundances showed fixation within a specific dilution range (0.005 to 0.1; Fig. 4 and Supplementary Fig. 6).

**Figure 5:**
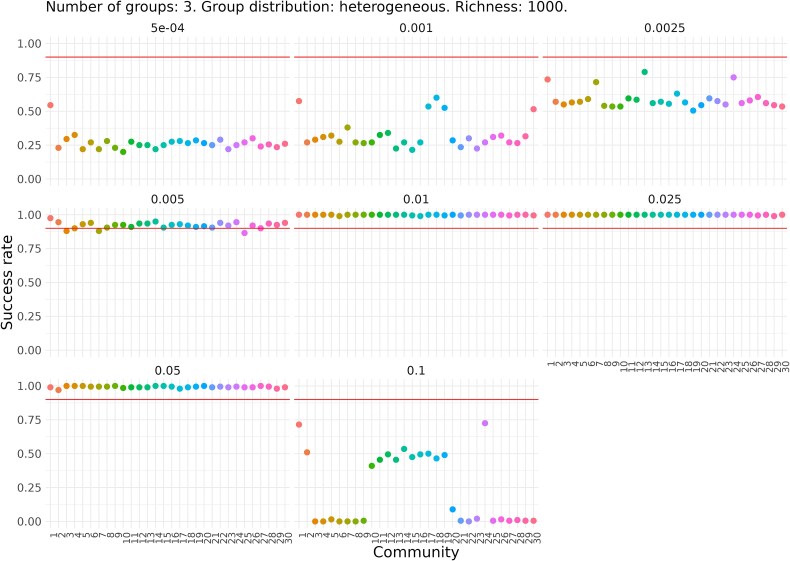
Success rate for each of the 30 simulated communities with 3 functional groups, heterogeneous relative abundances, and a richness of 1000. Each plot corresponds to a different dilution factor. The *Y* axis indicates the proportion (from 0 to 1) of dilution-growth simulations in which total success occurs; that is, fixation in all functional groups. Each point represents one of the 30 communities.

For communities with 3 functional groups, regardless of niche size distribution or richness, we observed a dilution factor range where success increases with dilution intensity. In communities with equal niche sizes, results were similar for both richness values (Supplementary Figs 7 and 8), between dilution factors 0.0025 and 0.05 all communities reached fixation of the three groups. However, at higher dilution intensities (0.001 and 0.0005) and the lowest dilution intensity (0.1), fixation occurred but with success rates below the threshold. The rise and then fall in success rates as dilution decreases suggests that weak dilutions may prevent larger groups from achieving fixation. For communities with variable niche sizes (Fig. 5 and Supplementary Fig. 9), the dilution range with success rates above 95% was narrower (0.005–0.05), likely because less abundant groups were more prone to extinction.

**Figure 6:**
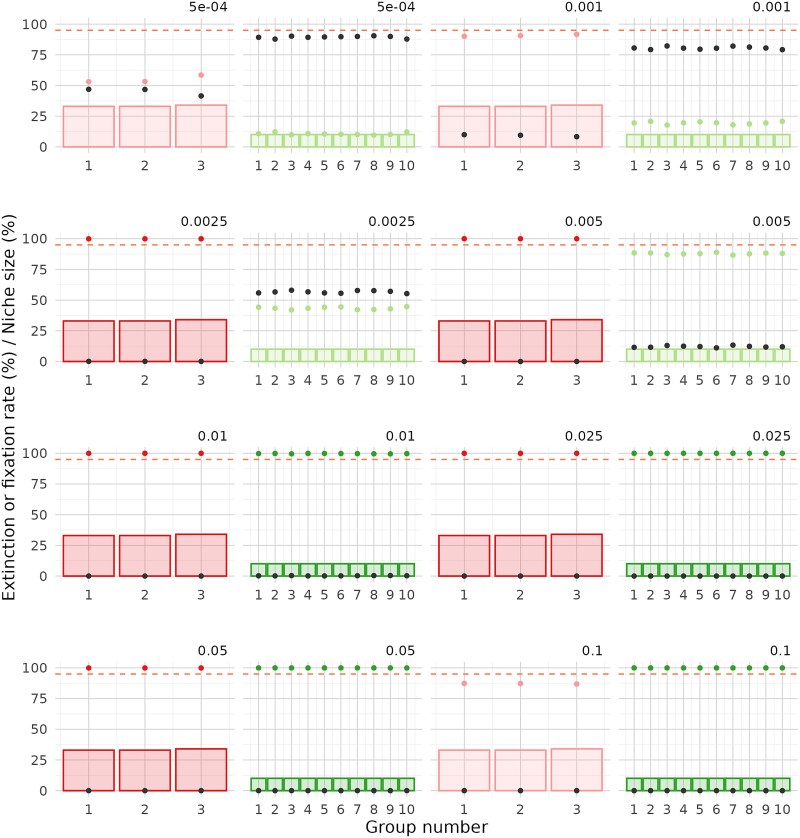
Fixation and extinction rate by functional group in communities with homogeneous niche abundances. Results are shown for communities with a richness of 100 and 3 or 10 groups (fixation in red and green, respectively, extinction in dark grey). Fixation for groups that exceed the 95% success threshold is highlighted in a darker shade. Bars represent the niche size associated with each functional group. Fixation is measured as the percentage of simulations in which at least one population from the group reaches fixation. Extinction is measured as the percentage of simulations in which no population from the group reaches fixation.

**Figure 7:**
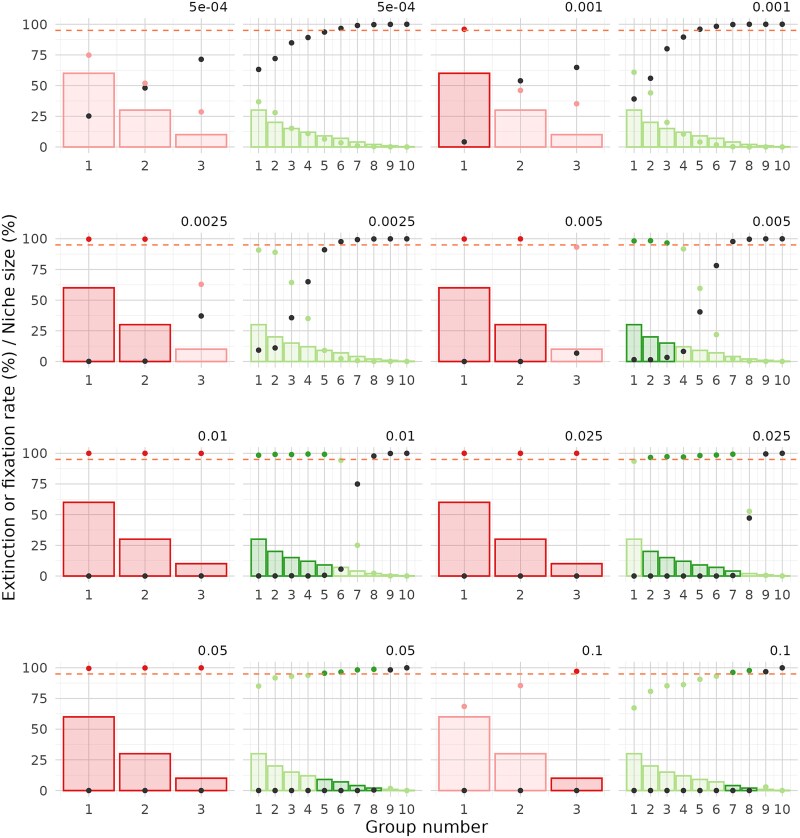
Fixation and extinction rate by functional group in communities with heterogeneous niche abundances. Results are shown for communities with a richness of 100 and 3 or 10 groups (fixation in red and green, respectively, extinction in dark grey). Fixation for groups that exceed the 95% success threshold is highlighted in a darker shade. Bars represent the niche size associated with each functional group. Fixation is measured as the percentage of simulations in which at least one population from the group reaches fixation. Extinction is measured as the percentage of simulations in which no population from the group reaches fixation.

Next, we examined the fixation rates of each individual group (Figs 6 and 7). As expected, in communities with homogeneous niche sizes, fixation of one group generally coincides with fixation of all groups. In communities with heterogeneous niche sizes, less intense dilution led to lower fixation rates in the larger groups compared to smaller ones. However, the two smallest groups were exceptions, showing near-zero fixation rates across all tested dilution factors, likely due to their higher extinction risk.

For communities with 3 groups, success was achieved for all groups at dilution factors between 0.01 and 0.05, regardless of richness. In communities with 10 groups, partial success was observed: fixation occurred in one or more groups but not simultaneously in all. For both richness levels (100 and 1000), the optimal dilution factors were 0.01 and 0.025, allowing up to 5 or 6 groups to exceed the 95% success threshold. To further investigate the observed effects, we examined the process from the perspective of group extinction (Figs 6 and 7 and Supplementary Figs 10 and 11). We found that the failure to achieve fixation in certain groups was mainly due to their extinction caused by excessively strong dilution factors, rather than an insufficient number of cycles. In summary, the results highlight a delicate balance between extinction and fixation among groups with both larger and smaller niche sizes.

**Figure 8:**
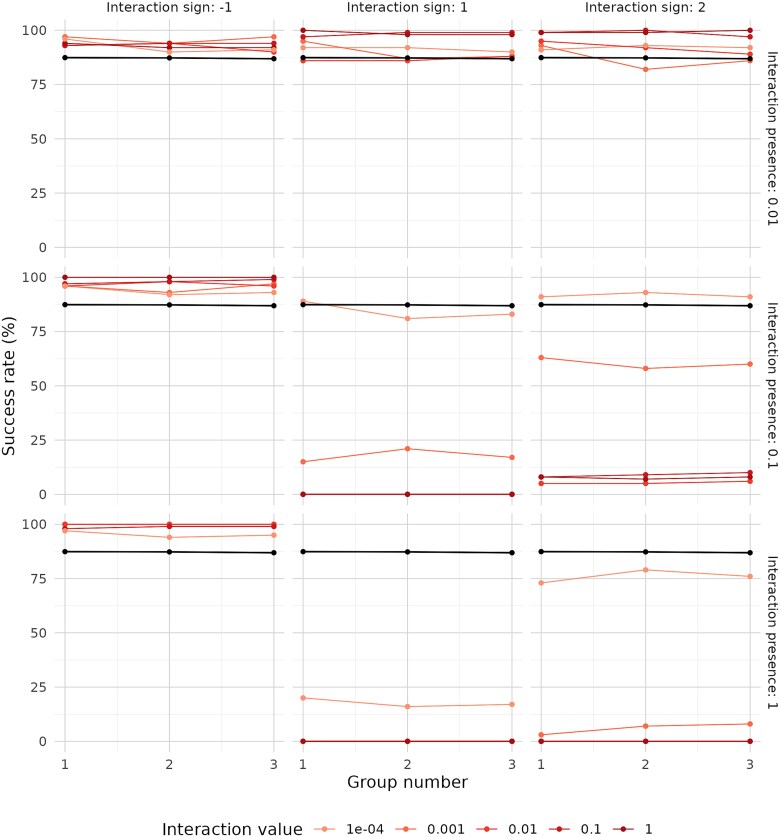
Success rate by functional group in communities with 3 groups (homogeneous group sizes, richness 100) following simulations under different interaction scenarios. Results without interactions are shown in black. Results for varying interaction strengths are displayed in different colors, indicating both the presence and the sign of the interaction. A dilution factor of 0.1 was used in all simulations.

Finally, we explored the potential effect of inter-group interactions (i.e. between populations from different functional groups) on the overall success of the strategy, as well as on the fixation and extinction of functional groups. For the community with 3 groups of equal niche size, we observed that infrequent interactions led to an increase in fixation rates, regardless of interactions sign (Fig. 8). The increase in fixation was more pronounced with positive interactions (either solely positive or mixed signs) at the two highest interaction strength values. This effect can be interpreted as an advantage for a limited number of populations over others. When interactions were exclusively negative, fixation rates improved as interaction frequency increased to 10% or 100%. One possible explanation is that these negative interactions promoted the extinction of some populations, thereby facilitating fixation of unaffected populations. However, this pattern did not hold when negative interactions had both an absolute strength of 1 and a 100% frequency; in this case, simulated growth was effectively suppressed. Finally, introducing strong and frequent positive interactions, whether alone or combined with negative ones, resulted in a sharp decline in fixation rates. This suggests a stabilization of a high number of intra-group populations, preventing fixation of any single population.

**Figure 9:**
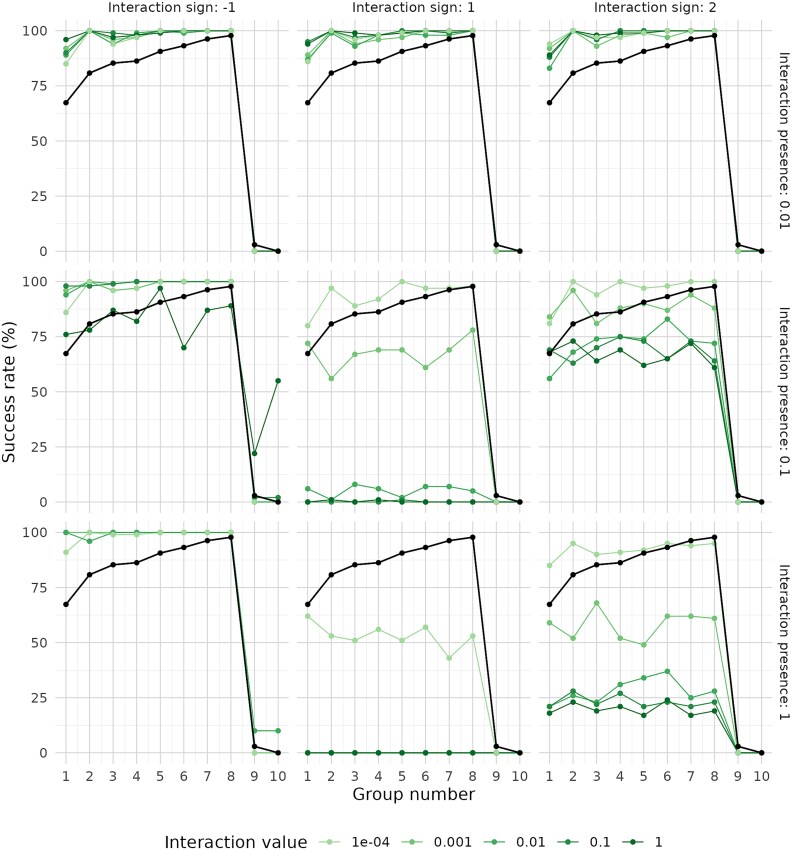
Success rate by functional group number in communities with 10 groups (heterogeneous group sizes, richness 100) after simulations under different interaction scenarios. Results without interactions are shown in black, while results under varying interaction strengths are shown in different colors, indicating both the presence and the sign of the interaction. A dilution factor of 0.1 was used in all simulations.

For the community with 10 functional groups of heterogeneous niche sizes, we again observed that negative interactions generally promoted higher fixation rates across all groups (Fig. 9). This effect was consistent across various combinations of interaction strength and frequency, though some exceptions were noted. In contrast, negative interactions with 100% frequency tended to suppress overall community growth. Once again, introducing positive interactions, either alone or in combination with negative ones, produced similar outcomes to infrequent negative interactions, as long as they remained rare. In other words, infrequent interactions consistently led to increased fixation rates, regardless of sign or absolute strength. On the other hand, simulations with positive interactions at medium to high frequencies generally resulted in a decrease in fixation rates, and this decrease was proportional to both interaction frequency and strength. The only exceptions occurred when the strength of positive interactions was low (0.0001) and either (i) the interaction frequency was 10%, or (ii) the frequency was 100% but included negative interactions as well. This suggests that positive interactions must remain limited for fixation rates to increase, mirroring the pattern observed in the three-group communities.

Additionally, while there was a clear positive correlation between group size and fixation in the simulations without interactions (with the exception of the two smallest groups, 9 and 10), the inclusion of interactions tended to reduce this correlation by leveling fixation rates across groups. However, the two smallest groups, being more vulnerable to extinction, generally did not benefit from negative interactions. Slight improvements in their fixation rates were observed only in two cases: weak negative interactions with 100% frequency (stronger interactions at this frequency were excluded due to growth suppression), and the strongest negative interactions with 10% frequency.

## Discussion

The parameter ranges explored in the simulations were chosen to broadly examine the conceptual space in which a drift-driven microbiome simplification strategy might operate, rather than to reproduce specific microbial ecosystems. In particular, the simulated community sizes (10^4^ and 10^6^ individuals) were selected to evaluate the behavior of the model across two markedly different population scales. These values may reasonably approximate small microbial ecosystems or rather small *in vitro* microbial communities, but they clearly remain far below the population sizes typically found in many natural microbial systems. In practice, 10^6^ also correspond to the largest community size that could be simulated within our current computational framework without incurring prohibitive runtimes given the hardware resources available to us.

Our results consistently reveal a clear relationship between community size and dilution factor, whose interaction largely determines the success of the proposed strategy. Larger communities would therefore be expected to require stronger dilution regimes in order to achieve comparable levels of drift-driven simplification. Similarly, the numbers of microbial populations and functional groups explored were intended to span different levels of community organization rather than to match specific ecosystems. Consistent with the simulation results, richness per se showed little influence on the outcome of the process. Instead, the key determinants appear to be the number of functional groups present and, more importantly, differences in their relative niche sizes. In particular, groups associated with smaller niches are more prone to extinction during repeated dilution–growth cycles, as discussed below.

Finally, with respect to the parameters used to model inter-population interactions, our aim was likewise to explore a broad range of possible interaction regimes rather than to reproduce specific ecological networks. Given the limited knowledge available on the structure and strength of microbial interaction networks in many systems, we explored wide ranges of interaction strengths and frequencies. As shown in the results and discussed below, the nature of these interactions can strongly influence the success of the proposed strategy by affecting fixation dynamics and the persistence of functional groups.

Beyond its practical implications for microbiome engineering, the present work provides a mechanistic view of how microbial communities assemble and stabilize under strong ecological drift. In this context, the serial dilution–growth process can be interpreted both as an experimental model of community assembly and as a form of global, approximately neutral mortality, a disturbance regime that is pervasive across microbial ecosystems, including aquatic flushing, intestinal transit, rainfall-driven leaf washing, soil percolation and shear-driven biofilm erosion.

Several recent studies have demonstrated that serial passaging, dilution-mediated mortality, dispersal and stochastic demographic processes can strongly reorganize microbial community composition and diversity in well-mixed systems, and that many of these effects can be captured by relatively simple theoretical frameworks [22–24]. These works have explored how drift, immigration, emigration and interspecific interactions shape microbial community dynamics and diversity patterns under serial passaging and dilution, and have also previously examined related aspects of these processes within similar experimental and theoretical frameworks.

Although the simulation framework used here cannot capture the full ecological complexity of natural microbial ecosystems, we previously demonstrated that the simulation framework employed here is capable of reproducing key patterns of microbial community composition and diversity observed in dilution–transfer experiments. In particular, in a previous study [17], this framework successfully recapitulated the diversity patterns observed in microbial communities organized around functional groups under serial dilution–transfer regimes. These results suggest that, despite its simplifying assumptions, the framework can capture relevant features of microbial community assembly and therefore provides a useful conceptual basis to explore the feasibility of drift-driven microbiome simplification.

Here, the study is centered on a distinct and complementary aim. Rather than focusing on predicting diversity patterns, phase transitions or neutrality–selection regimes, we specifically investigate the feasibility of exploiting drift-dominated passaging as a deliberate strategy for microbiome simplification and the generation of minimal, cohesive and functionally complete microbial consortia. By explicitly incorporating functional redundancy, functional groups and inter-group interactions into our simulation framework, we extend previous approaches toward an explicit end-point design problem, identifying combinations of ecological structure and experimental parameters that allow the fixation of non-redundant populations while preserving community function. In this sense, our results reposition serial passaging not only as a tool to probe microbial community dynamics, but also as a tractable framework for rational microbiome simplification and consortium design.

While the primary objective of this study was to assess the feasibility of a drift-dominated strategy for microbiome simplification, the same framework concomitantly provides insight into microbial community assembly under repeated stochastic disturbance. Although our analytical framework does not explicitly quantify community diversity, the outcome metrics considered here, specifically whether a single population becomes fixed within each functional group, provide a lower-bound proxy for population-level diversity. Under this interpretation, fixation corresponds to the extreme case of minimal intragroup diversity, whereas the absence of fixation indicates that diversity has not collapsed to a single population, without implying stable coexistence. Accordingly, while diversity per se was not directly analyzed, our results allow qualitative inferences regarding how drift, dilution intensity and inter-group interactions constrain diversity outcomes under repeated disturbance. Finally, although immigration is not considered in the present framework, the model captures a limiting assembly regime in which drift, dilution and inter-group interactions alone determine functional persistence and diversity constraints under repeated disturbance.

With respect to ecosystem function, our results indicate that the persistence of functional groups under repeated dilution-driven disturbance is primarily governed by dilution strength, which preferentially drives the loss of functional groups associated with smaller effective niche sizes. Superimposed on this effect, the structure of inter-group interactions further modulates functional persistence: communities in which interactions between functional groups are sparse and weak are more likely to retain all functional groups, whereas frequent or strong interactions, regardless of their sign, tend to promote the loss of entire groups. In this sense, the topology of the functional interaction network acts as a factor influencing functional completeness under chronic disturbance. Diversity outcomes primarily reflect the balance between stochastic drift, effective group size and dilution intensity, with stronger drift and higher dilution favoring fixation and reduced population-level diversity. At the same time, inter-group interactions can modify these diversity patterns indirectly: in particular, certain interaction structures prevent fixation within functional groups, thereby maintaining higher diversity, whereas other interaction regimes accelerate fixation or eliminate entire groups, resulting in reduced diversity.

Our simulations indicate that community size, dilution factor, and, to a lesser extent, initial community diversity, will significantly affect the success of the proposed strategy for microbiome simplification. There will be a delicate balance between the chosen dilution factor and the fixation or extinction of functional groups within the community, with marked differences between groups depending on their niche size. Furthermore, inter-group interactions will likely enhance the success of the approach when they are infrequent or weak (particularly if they are positive).

Based on our results, we conclude that while the successful implementation of the proposed approach for generating Minimal Microbiomes is feasible, it is constrained by several factors and will largely depend on the interplay between community size and dilution factor. However, even with an optimal balance between these parameters, functional groups with smaller niche sizes may still go extinct. Notably, the existence of inter-group interactions will likely increase the success of the approach when such interactions are either positive and infrequent, or negative but limited in frequency or intensity.

Therefore, in the practical applications of this process to generate Minimal Microbiomes, perfect outcomes should not be expected; some functional loss must be assumed as part of the process. Alternatively, one could apply less stringent dilution factors and accept limited fixation among the more abundant functional groups. Nonetheless, in the former case, the missing minority functional groups could potentially be recovered through a second iteration of the process. This would involve reapplying the approach using the previously obtained minimal microbiome co-inoculated with a small proportion of the original community. In doing so, larger niches would already be occupied by the minimal microbiome, and the parameters of the second experiment could be tuned to favor recovery of smaller functional groups. Although this solution would effectively double the experimental workload, it may be the only viable strategy for obtaining a truly complete Minimal Microbiome. In any case, the proposed approach offers a powerful tool for simplifying experimental microbiomes, significantly enhancing our ability to isolate key populations, thus facilitating the construction of low-diversity consortia that remain functionally comprehensive and ecologically cohesive.

## Supplementary Material

ycag067_Supplementary_Figures

## Data Availability

The data underlying this article are available in the GitHub repository accessible at https://github.com/silvtal/predicting_fixation.
