# Supplementary material for: Ecological drift simulations reveal key factors influencing minimal microbiome engineering and community assembly
Source: ISME Commun. 2026 Mar 21;6(1):ycag067. doi: 10.1093/ismeco/ycag067 (PMC13098171; doi:10.1093/ismeco/ycag067)
Supplement: ycag067_Supplementary_Figures [file ycag067_supplementary_figures.docx]

Supplementary Figures and Tables to “**Ecological drift simulations reveal key factors influencing minimal microbiome engineering and community assembly**”

Silvia Talavera-Marcos^1^, Daniel Aguirre de Cárcer^1*^

^1^Microbial and Environmental Genomics Group, Departamento de Biología, Universidad Autónoma de Madrid, Madrid, Spain.

**Supplementary Figure 1.** Reanalysis of Goldford et al. data showing progressive decrease of OTU richness along dilution–growth transfer cycles. Dynamics of richness across sequential transfer cycles for inocula 2 and 6 in glucose. Only these inocula were included because they presented sufficient sequencing depth in the original dataset across all transfer cycles to allow consistent richness estimation. Light gray shaded areas represent the interquartile range of richness. Individual community trajectories (colored lines) show a consistent reduction in OTU number across all replicates for both inocula.


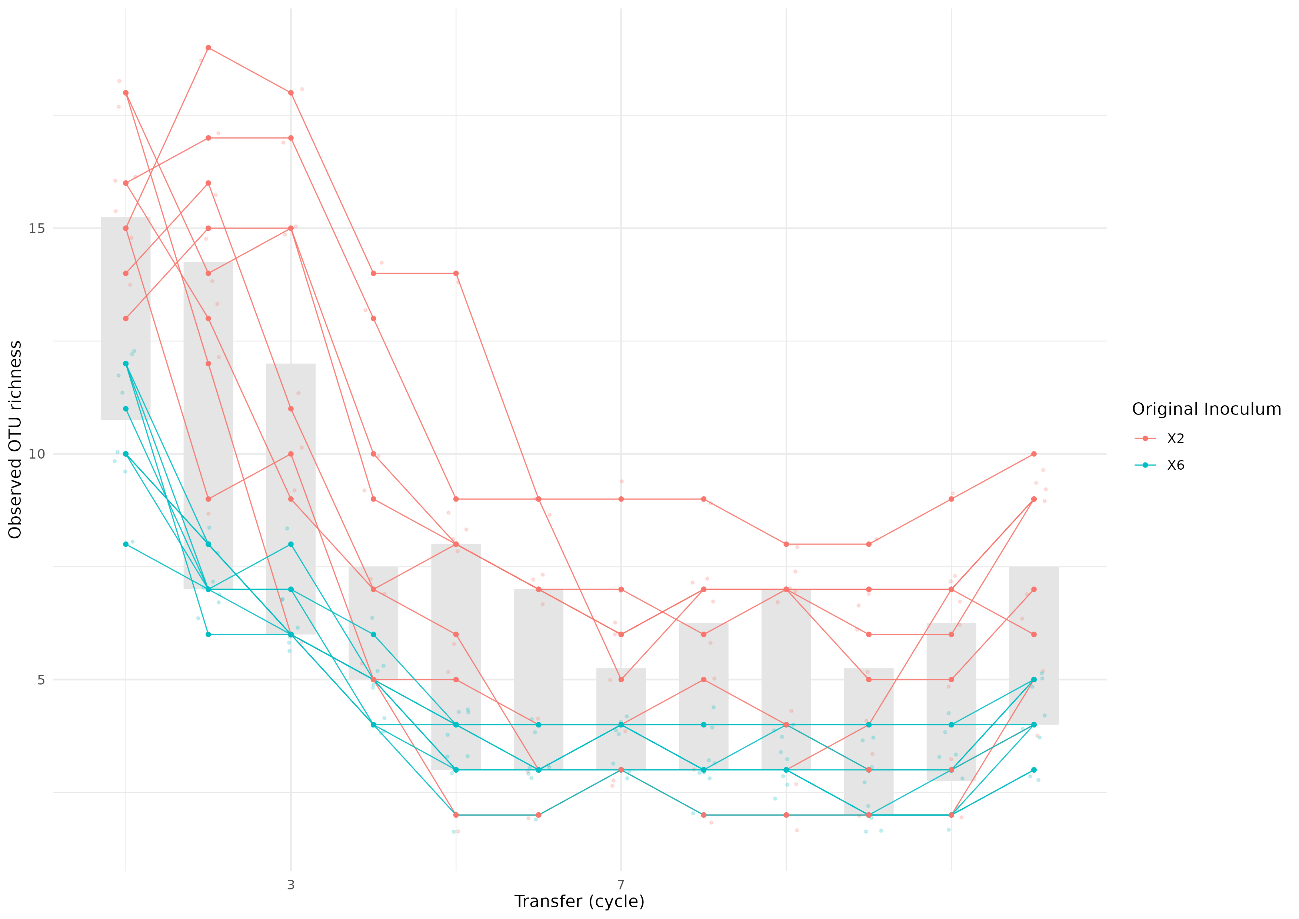


**Supplementary Figure 2**. Examples of abundances of four populations over time under three simulated scenarios: (A) without interactions, (B) with a negative interaction (P1 inhibits P3), and (C) with a positive interaction (P1 promotes the growth of P3). Solid lines represent the populations of Functional Group 1 (P1 and P2, carrying capacity 60%), while dashed lines represent the populations of Functional Group 2 (P3 and P4, carrying capacity 40%). Lines of different colors represent the abundance of each population over 10 successive dilution-growth cycles.


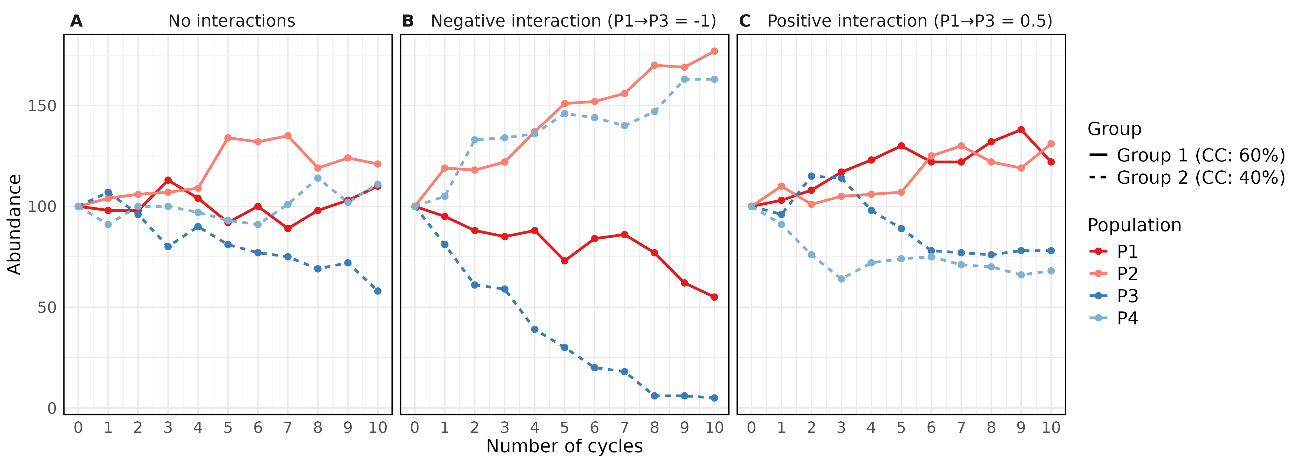


**Supplementary Figure 3**. Success patterns of dilution-growth processes according to the simulation parameters used. The Y axis indicates the dilution-growth cycle where success occurred, while the X axis orders the different simulated communities by dilution factor, distribution, community size, and richness. Each experiment is colored according to community type (same values of community size, abundance distribution, and richness); color intensity represents the dilution factor. Left; fixation threshold of 90%, Right; fixation threshold of 50%.


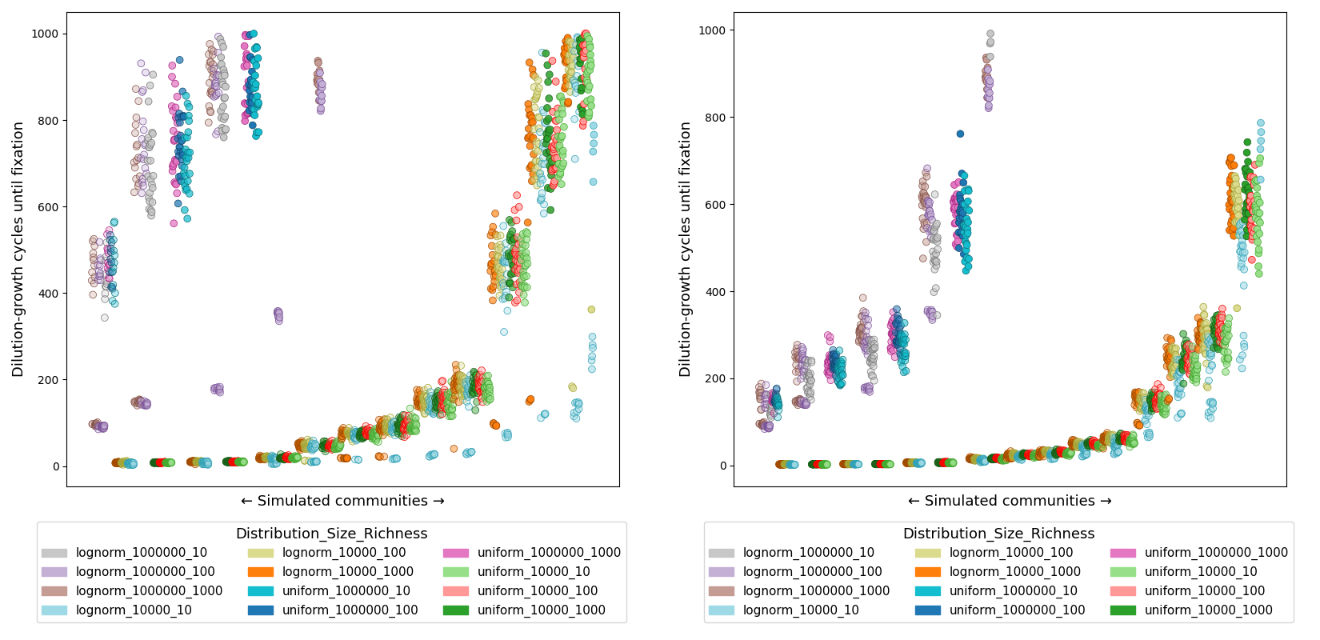


**Supplementary Figure 4**. Success rate for each of the 30 simulated communities with 10 functional groups, heterogeneous relative abundances, and a richness of 1000. Each plot corresponds to a different dilution factor. The Y axis indicates the proportion (from 0 to 1) of dilution-growth simulations in which total success occurs; that is, fixation in all functional groups. Each point represents one of the 30 communities. The missing point corresponds to a community that, after experiencing extinctions and a drop in total abundance, could not be sustained under the applied dilution factor.


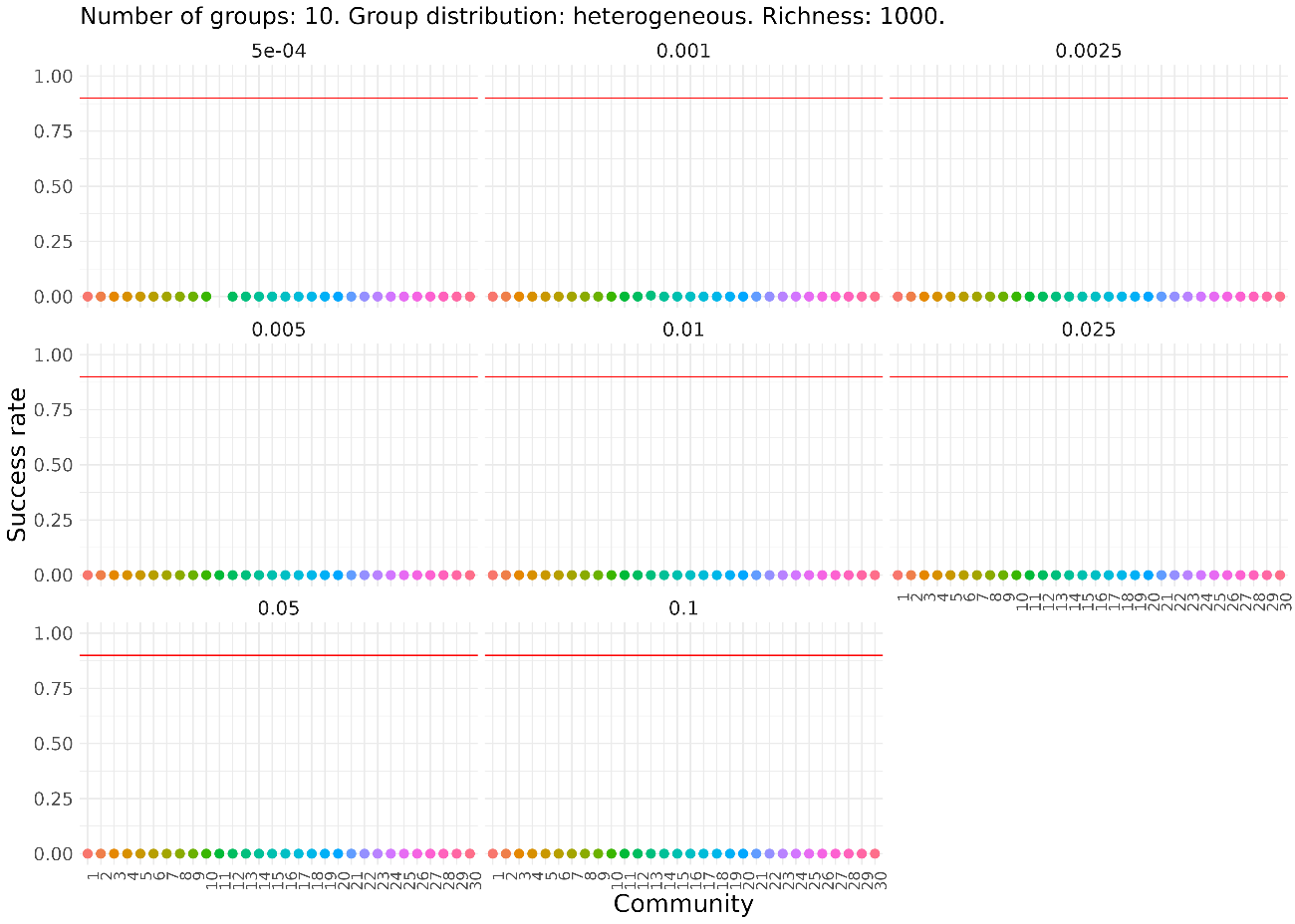


**Supplementary Figure 5**. Success rate for each of the 30 simulated communities with 10 functional groups, heterogeneous relative abundances, and a richness of 100. Each plot corresponds to a different dilution factor. The Y axis indicates the proportion (from 0 to 1) of dilution-growth simulations in which total success occurs; that is, fixation in all functional groups. Each point represents one of the 30 communities.


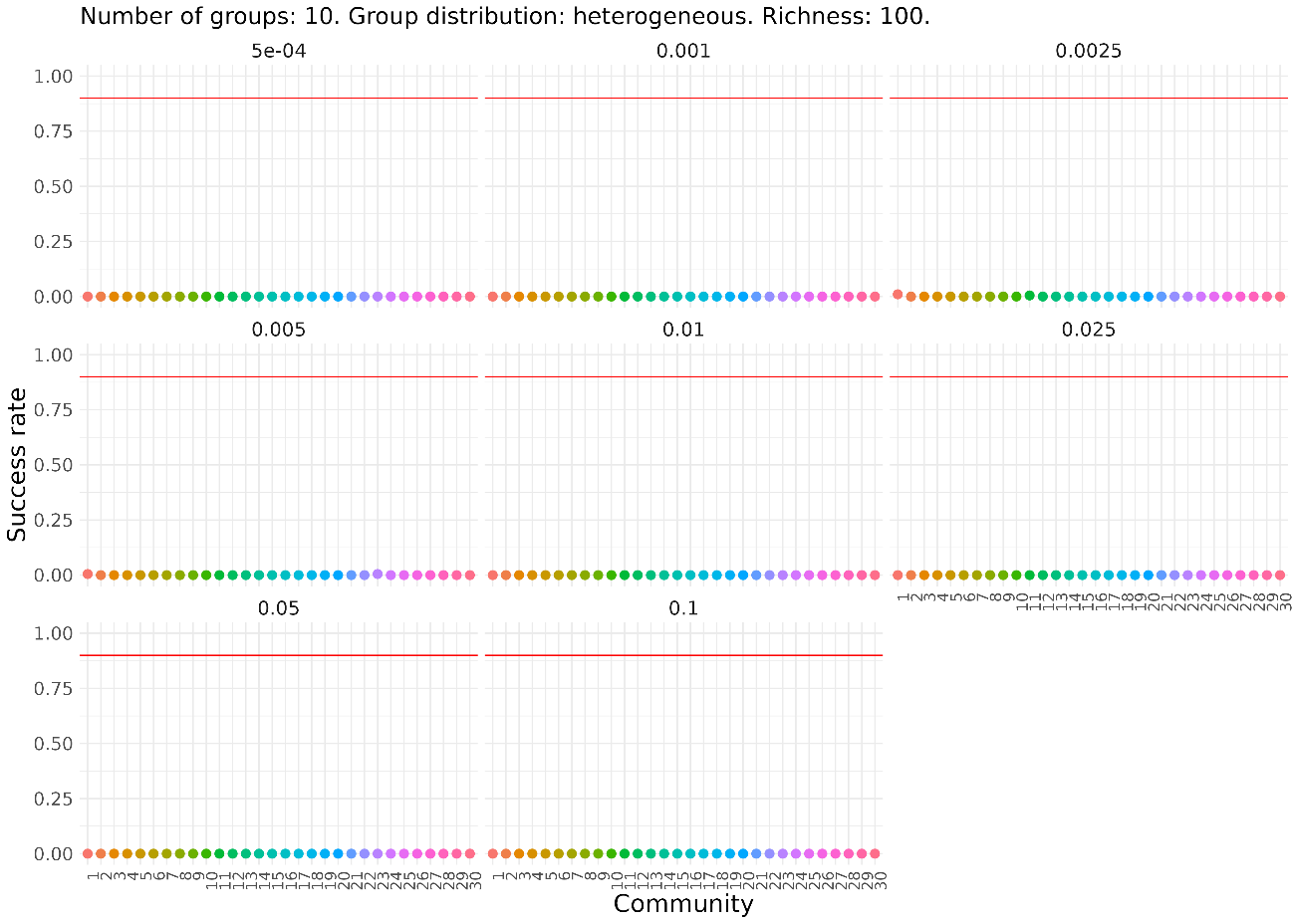


**Supplementary Figure 6**. Success rate for each of the 30 simulated communities with 3 functional groups, homogeneous relative abundances, and a richness of 1000. Each plot corresponds to a different dilution factor. The Y axis indicates the proportion (from 0 to 1) of dilution-growth simulations in which total success occurs; that is, fixation in all functional groups. Each point represents one of the 30 communities.


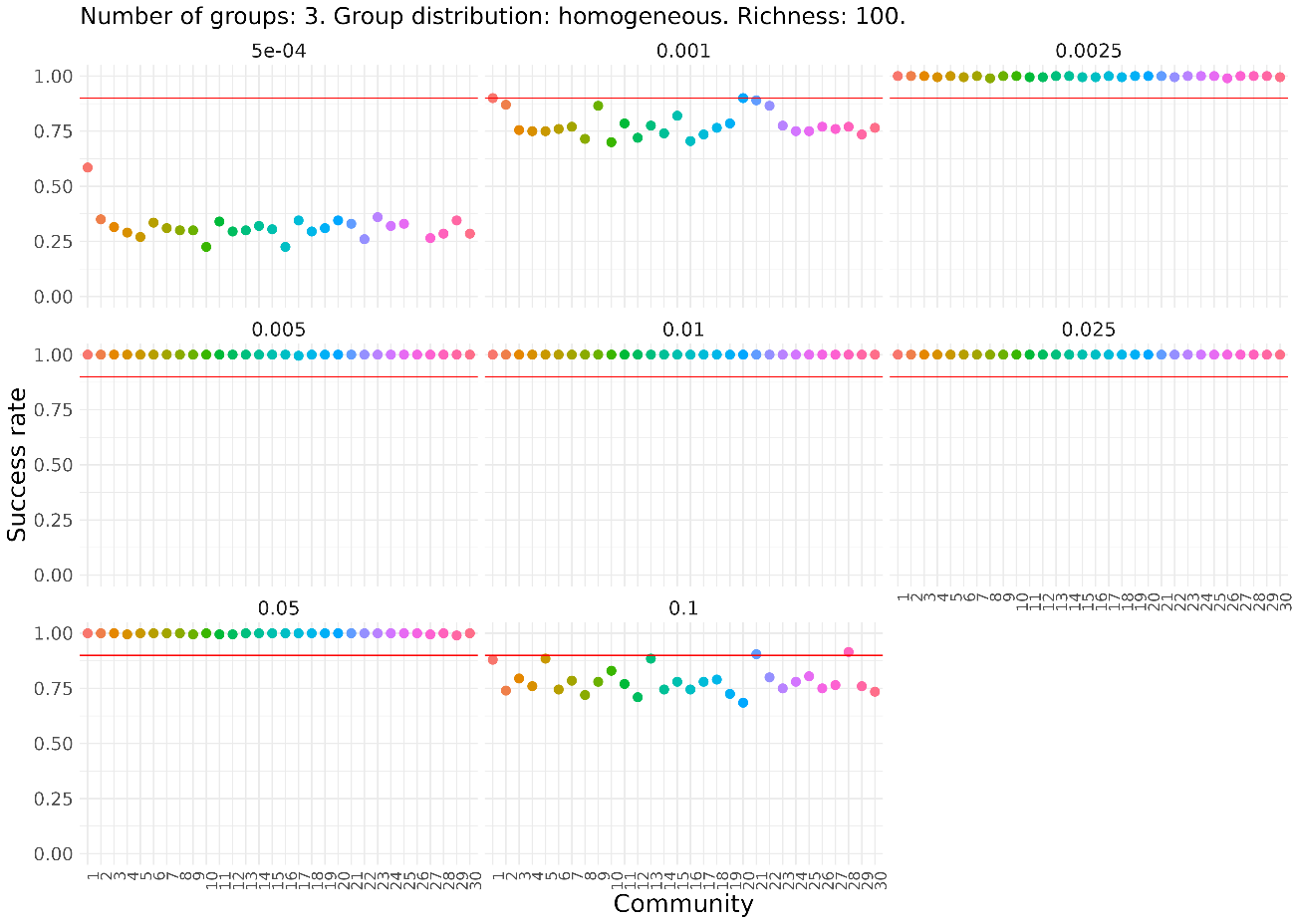


**Supplementary Figure 7**. Success rate for each of the 30 simulated communities with 3 functional groups, homogeneous relative abundances, and a richness of 100. Each plot corresponds to a different dilution factor. The Y axis indicates the proportion (from 0 to 1) of dilution-growth simulations in which total success occurs; that is, fixation in all functional groups. Each point represents one of the 30 communities.


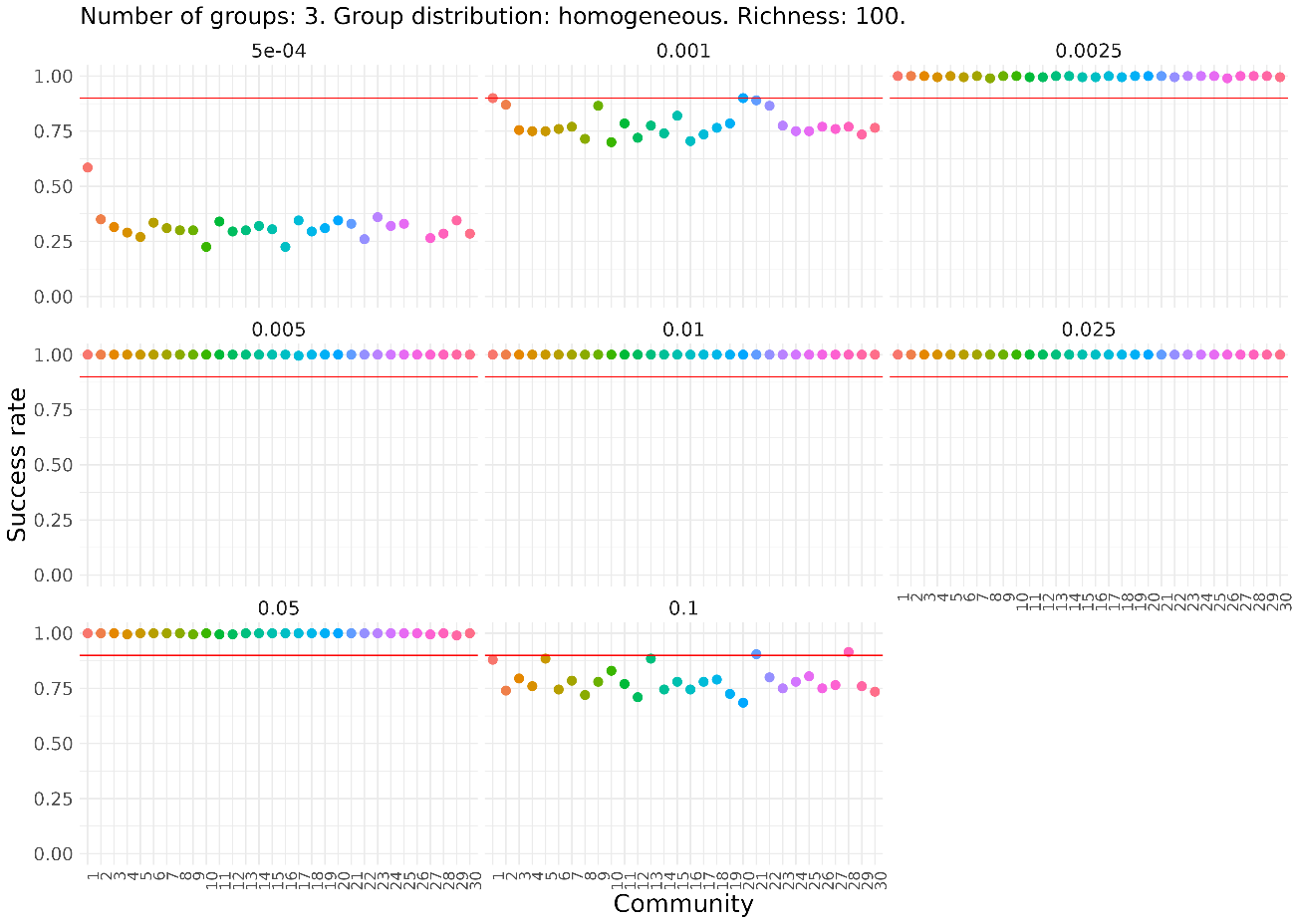


**Supplementary Figure 8**. Fixation and extinction rate by functional group in communities with homogeneous niche abundances. Results are shown for communities with a richness of 1000 and 3 or 10 groups (fixation in red and green, respectively, extinction in dark grey). Fixation for groups that exceed the 95% success threshold is highlighted in a darker shade. Bars represent the niche size associated with each functional group. Fixation is measured as the percentage of simulations in which at least one population from the group reaches fixation. Extinction is measured as the percentage of simulations in which no population from the group reaches fixation.


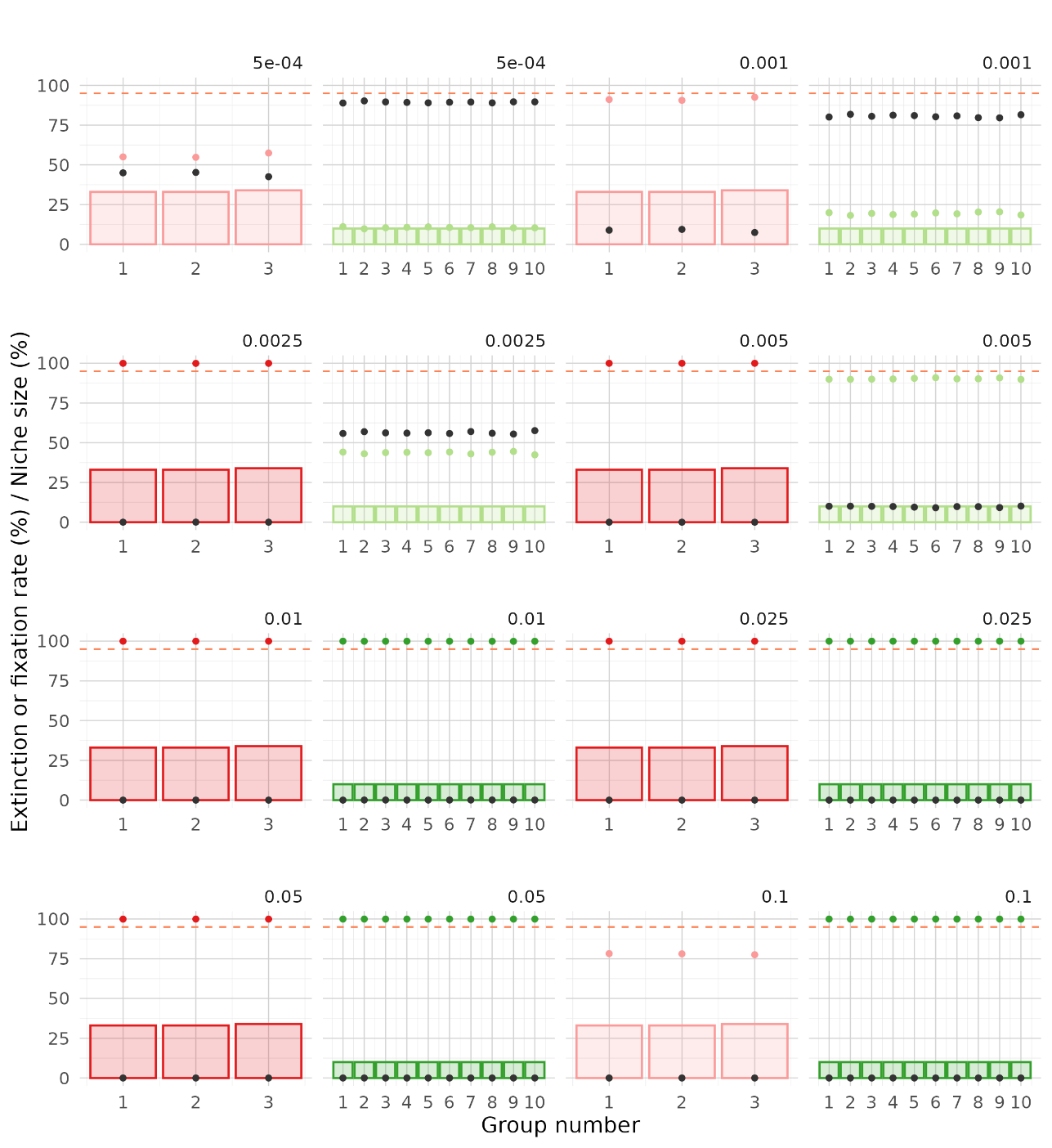


**Supplementary Figure 9**. Fixation and extinction rate by functional group in communities with heterogeneous niche abundances. Results are shown for communities with a richness of 1000 and 3 or 10 groups (fixation in red and green, respectively, extinction in dark grey). Fixation for groups that exceed the 95% success threshold is highlighted in a darker shade. Bars represent the niche size associated with each functional group. Fixation is measured as the percentage of simulations in which at least one population from the group reaches fixation. Extinction is measured as the percentage of simulations in which no population from the group reaches fixation.


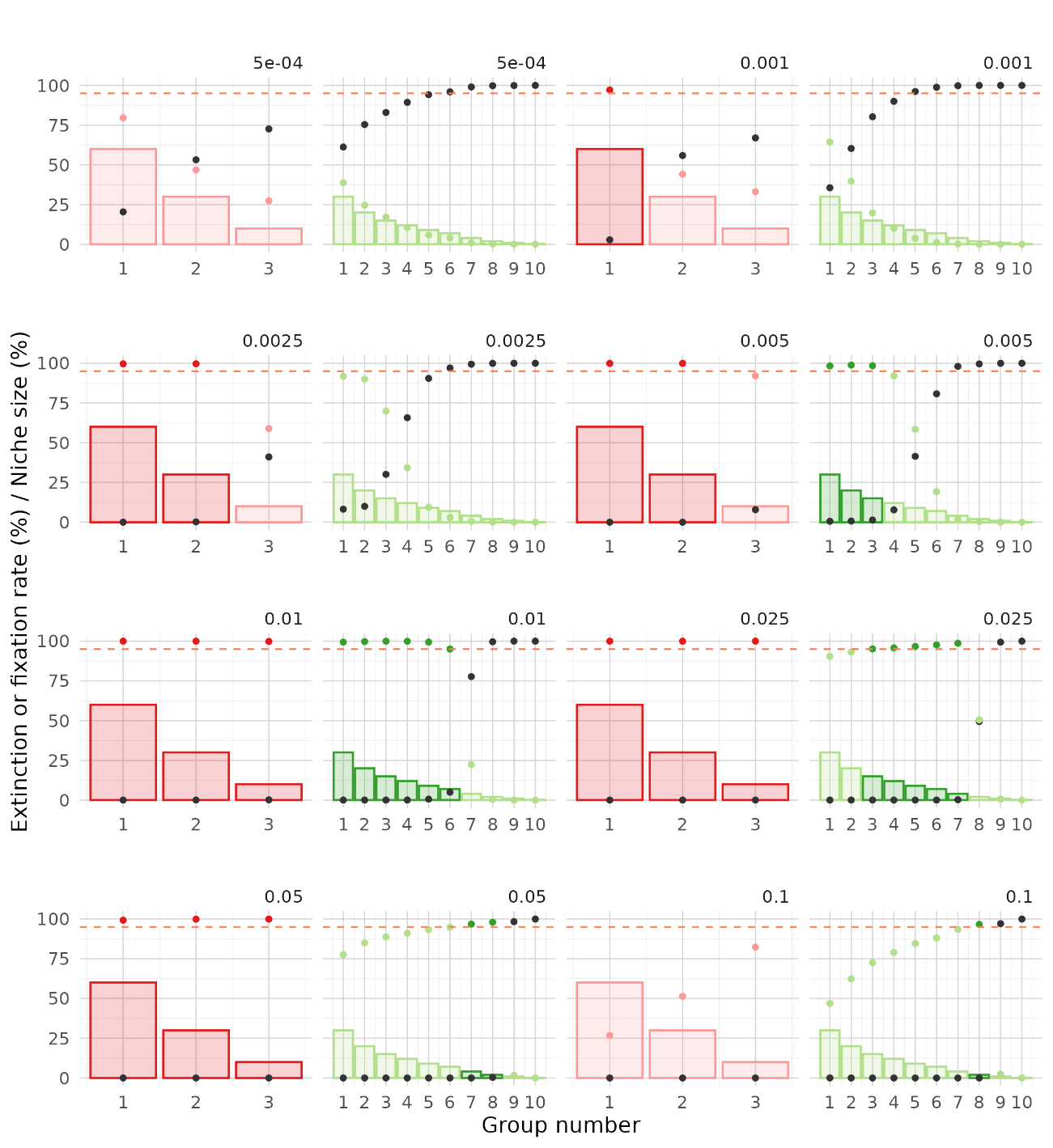


**Supplementary Table 1**. Mean decrease in accuracy for each variable used in the following models: (1) Pielou’s evenness only, (2) Shannon diversity only, (3) Gini index only, (4) Evenness and richness, (5) Shannon diversity and richness, (6) Gini index and richness. Success is defined using a fixation threshold of 50%.

| Model | Community size | Dilution factor | Pielou’s evenness | Shannon diversity | Gini index | Richness |
| --- | --- | --- | --- | --- | --- | --- |
| 1 | 0.576 | 0.509 | 0.033 | NA | NA | NA |
| 2 | 0.573 | 0.509 | NA | 0.038 | NA | NA |
| 3 | 0.575 | 0.509 | NA | NA | 0.032 | NA |
| 4 | 0.574 | 0.509 | 0.029 | NA | NA | 0.024 |
| 5 | 0.573 | 0.509 | NA | 0.039 | NA | 0.014 |
| 6 | 0.574 | 0.509 | NA | NA | 0.029 | 0.035 |

**Supplementary Table 2**. Mean decrease in accuracy for each variable used in the following models: (1) Pielou’s evenness only, (2) Shannon diversity only, (3) Gini index only, (4) Evenness and richness, (5) Shannon diversity and richness, (6) Gini index and richness. Success is defined using a fixation threshold of 90%.

| Model | Community size | Dilution factor | Pielou’s evenness | Shannon diversity | Gini index | Richness |
| --- | --- | --- | --- | --- | --- | --- |
| 1 | 0.540 | 0.52 | 0.072 | NA | NA | NA |
| 2 | 0.539 | 0.52 | N | 0.091 | NA | NA |
| 3 | 0.537 | 0.52 | NA | NA | 0.083 | NA |
| 4 | 0.539 | 0.52 | 0.073 | NA | NA | 0.056 |
| 5 | 0.539 | 0.52 | NA | 0.083 | NA | 0.064 |
| 6 | 0.539 | 0.52 | NA | NA | 0.081 | 0.052 |

**Supplementary Table 3**. R² values for each of the following models, defined according to the parameters they include: (1) Pielou’s evenness only, (2) Shannon diversity only, (3) Gini index only, (4) Evenness and richness, (5) Shannon diversity and richness, (6) Gini index and richness.

| Fixation threshold | 1 | 2 | 3 | 4 | 5 | 6 |
| --- | --- | --- | --- | --- | --- | --- |
| 50% | 0.990 | 0.991 | 0.990 | 0.991 | 0.991 | 0.991 |
| 90% | 0.929 | 0.936 | 0.934 | 0.937 | 0.937 | 0.93 |
